# Inflammation: The Beauty or the Beast? Vitamins, Nutritional Supplements, Antioxidant Therapy, and Modulators of Inflammation as Therapeutic Interventions

**DOI:** 10.3390/nu16213630

**Published:** 2024-10-25

**Authors:** Daniela Caporossi, Antonio Herbert Lancha, Dario Coletti

**Affiliations:** 1 Unit of Biology and Genetics of Movement, Department of Movement, Human and Health Sciences, University of Rome Foro Italico, Piazza Lauro De Bosis 15, 00135 Rome, Italy; daniela.caporossi@uniroma4.it; 2 Experimental Surgery (LIM 26), Laboratory of Clinical Investigation, School of Medicine, University of Sao Paulo, Avenida Doutor Arnaldo 455, São Paulo 05508-030, SP, Brazil; lanchajr@usp.br; 3 Unit of Histology and Medical Embryology, Department of Anatomy, Histology, Forensic Medicine and Orthopedics, Sapienza University of Rome, 00161 Roma, Italy; 4 Biological Adaptation and Ageing (B2A), Institut de Biologie Paris-Seine, Sorbonne Université, CNRS UMR 8256, Inserm U1164, 75005 Paris, France

The importance of inflammation in disease development is now well known not only for acute states but also for chronic pathologies. Inflammation may be the beauty or the beast of an injury or a chronic disease state, turning the evolution of damage into heaven or hell, as does the “She” in Kretzmer/Aznavour’s immortal song*. A greater understanding of the mechanisms underlying inflammation and advancements in the use of inflammation modulators is of pivotal importance for interventions aimed at prevention or therapy. Various agents have the potential to regulate inflammation by activating specific signaling pathways [1,2]. Vitamins are essential micronutrients, with a well-established role in inflammation and metabolism. In some cases, the beneficial effects of vitamins stem from their antioxidant capacity and can be provided by antioxidant pharmacological treatments. Additionally, numerous natural compounds have these properties and are currently being explored as immune modulators. Indeed, targeting oxidative stress has been proposed for the treatment or management of major pathological disease states, from diabetes to obesity and from hypertension to cachexia. The most innovative approaches in medicine, such as multimodal interventions and tailored medicine, currently include nutritional supplementation to buffer the redox status of the body or to naturally trigger beneficial signaling pathways to control inflammation and metabolic alterations. This *Nutrients* Special Issue is dedicated to this topic (https://www.mdpi.com/journal/nutrients/special_issues/Vitamins_Supplements_Antioxidant_inflammation_Therapeutic_Interventions accessed on 15 October 2024), and comprises a collection of papers proposing the use of vitamins, nutrients, and other factors for this purpose, expanding the margins of intervention for disease states characterized by inflammation.

## 1. An Overview of the Published Articles

When working with plant/fungi extracts, the standardization of the production process and its quality control, the bioinformatic analysis of the potential biological activities of the compounds, and the experimental validation of their efficacies are critical issues. Nakatake et al. report that a standardized extract of cultured Lentinula edodes mycelia (ECLM, AHCC^®^) has protective effects against liver damage caused by ischemia–reperfusion injury and partial hepatectomy in rodents (contribution 1). In this study, the authors also report various mechanisms underlying the beneficial effects of ECLM. These include the suppression of inflammatory responses through the increase in IL-10, and increased liver regeneration through the promotion of hepatocyte proliferation and the suppression of apoptosis (contribution 1). Colomba et al. show that the quercetin derivatives present in Prunus spinosa fruit extract exert antioxidant, antimicrobial, and anti-inflammatory activities, with the potential to promote wound healing (contribution 3). The authors show quercetin internalization by the human monocyte U937 cell line, resulting in the increased nuclear activity of Nuclear factor E2-related factor 2 (contribution 3).

Several vitamins and their metabolites have the potential to control cell damage and survival, as well as being able to modulate inflammation, which in turn exacerbates cell damage. Gao et al. use both in vitro and in vivo models of ulcerative colitis to explore the beneficial effects of vitamin D treatment (contribution 2). In particular, they show the presence of typical markers of inflammation, oxidative stress and ferroptosis, in these models and demonstrated that vitamin D and iron homeostasis contribute to the buffering of cell stress and death (contribution 2). Three research groups deal with the management of gut dysfunctional states through the use of vitamins and other factors. Li et al. show that vitamin A ameliorates diarrhea in a piglet model, not only reducing diarrhea incidence but also attenuating enteric glial gliosis and inflammatory responses, including immune cell infiltration (contribution 4). Ultimately, Li et al. conclude that vitamin A has a protective effect on the intestinal barrier through a retinoic acid-dependent pathway (contribution 4). In the chronic gut disease condition irritable bowel syndrome, Xie et al. pinpoint the role of vitamin D, along with calcium and parathyroid hormone (contribution 5). The study does not show any relationship between the serum levels of calcium and vitamin D and the risk of irritable bowel syndrome; however, a significant association with the parathyroid hormone level is reported (contribution 5). Interestingly, Hu et al. show that abdominal pain in a rat model of inflammatory bowel disease—which shares some symptoms with the aforementioned irritable bowel syndrome and is characterized by chronic inflammation—is alleviated by electrolyzed hydrogen water (contribution 6). The latter, also known as electro-activated water, is produced by the electrolysis of tap water and has free-radical-scavenging ability and disinfectant properties. Hu et al. find that it diminishes intestinal inflammation, ultimately alleviating abdominal pain (contribution 6).

An ex post study by Qiao et al. on patients with acute respiratory distress syndrome enrolled in a randomized, placebo-controlled trial focuses on the effects of high doses of intravenously delivered vitamin C (contribution 7). The authors find that vitamin C infusion diminishes the biomarkers of NETosis, i.e., the activation and release of neutrophil extracellular traps, and endothelial glycocalyx degradation; ultimately, this affects lung oxygenation and mortality (contribution 7).

A systematic review on Coenzyme Q10 supplementation protocols for periodontitis therapy by Merle et al. concludes this Special Issue (contribution 8). Coenzyme Q10, the endogenous component of the mitochondria, plays a key role in energy production, and has also antioxidant and anti-inflammatory properties; it can be delivered to the gingiva by topical administration or systemically through a dietary supplement. In this review, the authors systematically collect details of studies on Coenzyme Q10 as an adjunct to non-surgical periodontitis therapy and provide recommendations for possible clinical protocols (contribution 8).

**Figure 1:**
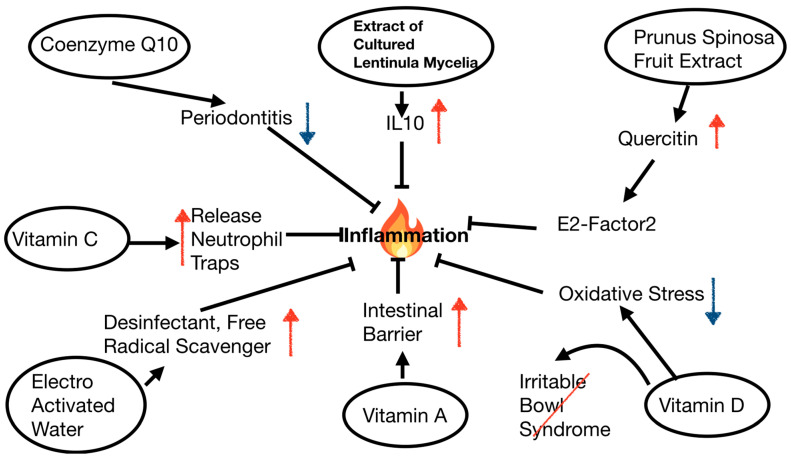
Major factors proposed as a treatment for various disease states share the ability to modulate inflammation.

The common denominator of all the reported treatments and pathways was a reduction in inflammation, highlighting the importance of controlling this process, in both acute and chronic conditions (Figure 1).

## 2. Conclusions

Functional nutrients are components of food that are exploited for their health benefits [3]. They include proteins/amino acids (e.g., branched-chain amino acids), lipids (e.g., polyunsaturated fatty acids), vitamins and their derivatives (e.g., vitamin D, Trolox), plant-derived compounds (e.g., curcumin, quercetin), and pre- and pro-biotics, i.e., compounds favoring the growth of intestinal microbiota or the microorganisms themselves, respectively [4]. The concept of functional nutrients to intervene not only with metabolism [5] but also with disease states [6] has received significant attention and their use in clinical practice is now gaining momentum. Striking examples of the use of functional nutrients for severe conditions, such as neurogenic muscle atrophy, are reviewed and discussed by Moresi et al. [7].

Oxidative stress due to oxygen/nitrogen free radicals present at a level beyond what is physiologically acceptable is associated with many acute and chronic diseases and aging [8]. Oxidative stress directly damages cell components and effects DNA repair and cell proliferation, in turn regulating gene expression [9]. Many of the aforementioned functional nutrients, such as quercetin, act directly on oxidative stress [10] and are proposed for the treatment of age- and disease-associated damage [11]. Examples of these approaches have been collected in this Special Issue. Something worth noting is that physical activity is another systemic intervention with enormous therapeutic potential [12,13] through the modulation of the redox status and inflammation [14,15], but this type of intervention has not been included in the present Special Issue and has been reviewed elsewhere [16]. Oxidative stress and chronic inflammation are tightly linked and are the triggers of major disease states, such as cancer [17], hypertension [18], and diabetes [19].

In conclusion, inflammation modulators that act through the control of the redox state offer countless opportunities for treatment. Many of these modulators can be introduced into organisms through functional nutrients and include vitamins and other supplements, characterized by their antioxidant properties. The study of biomarkers of oxidative stress and inflammation and the design of novel, powerful interventions will greatly improve the prognosis the management of major diseases and comorbidities.

* “She” (1974), written by Charles Aznavour and Herbert Kretzmer, label Barclay:

She may be the beauty or the beast
May be the famine or the feast
May turn each day into a heaven or hell
She may be the mirror of my dream
A smile reflected in a stream
She may not be what she may seem inside her shell
